# Probucol enhances the therapeutic efficiency of mesenchymal stem cells in the treatment of erectile dysfunction in diabetic rats by prolonging their survival time via Nrf2 pathway

**DOI:** 10.1186/s13287-020-01788-3

**Published:** 2020-07-21

**Authors:** Haoran Wang, Keqin Zhang, Zheng Ruan, Dingqi Sun, Hui Zhang, Guiting Lin, Liangliang Hu, Shengtian Zhao, Qiang Fu

**Affiliations:** 1 Department of Urology, Shandong Provincial Hospital, Cheeloo College of Medicine, Shandong University, Jingwuweiqi Road 324#, Jinan, 250021 Shandong People’s Republic of China; 2 Department of Urology, Shandong Provincial Hospital Affiliated to Shandong First Medical University, Jinan, 250021 Shandong People’s Republic of China; 3 Tai’an City Central Hospital, Tai’an, 271000 People’s Republic of China; 4 Knuppe Molecular Urology Laboratory, Department of Urology, School of Medicine, University of California, San Francisco, CA USA; 5 Department of Urology, Shandong Zaozhuang Municipal Hospital, Zaozhuang, 277000 People’s Republic of China

**Keywords:** Mesenchymal stem cell, Probucol, Oxidative stress, Nrf2, Autophagy, Apoptosis

## Abstract

**Background:**

Intracavernous injection of mesenchymal stem cells (MSCs) is a promising method for diabetic mellitus-induced erectile dysfunction (DMED), but short survival time of MSCs in cavernous is a fatal defect for therapy. This study investigated therapeutic efficiency and potential mechanism of probucol combined with MSCs.

**Methods:**

In vivo study, a total of forty-eight 10-week-old male Sprague-Dawley (SD) rats were used. Twelve rats received intraperitoneal injection of PBS as the sham group; the rest received intraperitoneal injection of 60 mg/kg streptozotocin to establish DM models. DM rats were randomly divided into three groups: received intracavernosal (IC) injection of either PBS (DM group), MSCs (M group), or administrated probucol after intracavernosal injection of MSCs (P + M group). Erectile function was assessed by electrical stimulation of the cavernous nerves with real-time intracavernous pressure measurement. After euthanasia, penile tissue was investigated for histologic examination and Western blotting. In in vitro experiment, H_2_O_2_ was used to create oxidative stress environment to detect changes in cell viability. CCK8 was used to measure cell viability of MSCs treated with or without probucol. Intracellular ROS changes were detected by flow cytometry. Autophagy and apoptosis were detected by Western blotting and confocal microscopy.

**Results:**

Recovery of erectile function was observed in the P + M group. The combination therapy decreased fibrosis and increased endothelial function compared with MSC therapy alone. Western blotting results confirmed the increased expression of Nrf2 and HO-1 in cavernous body. H_2_O_2_ induced high oxidative stress and reduced cell viability in vitro, which was gradually reversed with increased concentration of probucol. H_2_O_2_ reduced Nrf2 expression, which was reversed by probucol’s intervention. Furthermore, the expression of Bax, Caspase3, and Cleaved-Caspase3 decreased, and the expression of Bcl-2 increased in a dose-dependent manner because of probucol’s intervention. In addition, Beclin1 and LC3II both increased in a dose-dependent manner. Meanwhile, the expression of P62 decreased. In the study of autophagy flux, we found probucol did not block it.

**Conclusion:**

Probucol enhanced therapeutic efficiency of MSCs in DMED by prolonging their survival time, which mediated through improving the transplanted microenvironment of MSCs, increasing self-antioxidant ability of MSCs, strengthening protective autophagy, and inhibiting apoptosis of MSCs via Nrf2 pathway.

**Graphical abstract:**

Schematic model showing combined probucol and MSCs to improve DMED. Probucol increases self-antioxidant ability of MSCs, strengthening protective autophagy and inhibiting apoptosis via Nrf2/HO-1 and Nrf2/autophagy pathways. 
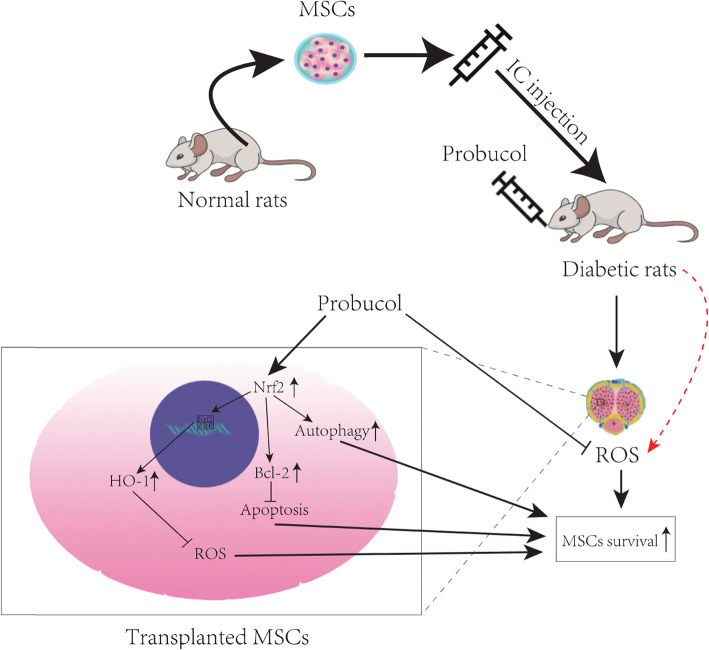

## Background

Erectile dysfunction (ED) is the consistent or recurrent inability to achieve and/or maintain a penile erection sufficient for satisfactory sexual performance [1]. Over the past few years, erectile dysfunction (ED) is a common complication in patients with diabetes mellitus (DM) [2]. Up to now, the mechanism of DMED is not fully understood. Increasing pieces of evidence show that oxidative stress is considered a major factor in the promotion and development of DMED [3]. Our previous studies confirmed that the DMED could be improved by attenuating oxidative stress.

At present, phosphodiesterase type 5 inhibitors (PDE5i) are the first line treatment for ED. But its effect on DMED is disappointing [4, 5]. Intracavernous injection of mesenchymal stem cells (MSCs) is a novel therapy. It has been proved that intracavernous injection of MSCs could significantly improve DMED in preclinical research and human clinical trials [6, 7]. The fatal defect of MSC therapy is the short maintenance time of curative effect, which is due to the short survival time of MSCs in the corpus cavernosum of the penis. In the corpus cavernosum of diabetic rats, the increase of reactive oxygen species (ROS) inhibits MSC proliferation, increases senescence, decreases differentiation, and inhibits MSC immune regulation [8]. Therefore, how to improve the activity and survival time of transplanted MSCs become urgent problems in DMED therapy.

Probucol has been used as anti-atherosclerotic for many years for its affection on anti-inflammatory and anti-oxidative. According to these pharmacological activities, probucol has been used in the treatment of DMED [9, 10]. Our previous study found that probucol can improve erectile function in diabetic rats, which may be related to activation of Nrf2 pathway [11].

Many studies show that Nrf2 plays a protective role in many pathological processes, including DMED [11, 12]. Nrf2 is a basic leucine zipper REDOX sensitive transcription factor that can regulate the REDOX state of cells when exposed to harmful stimuli [13]. Under basic condition, Nrf2 locates in the cytoplasm with its anchor inhibitors Kelch sample ECH associate protein 1 (Keap1) [14]. However, under oxidative stress, Nrf2 disintegrates from Keap1, relocates to the nucleus, and regulates downstream oxidation resistance genes. The activation of Nrf2 was found to have anti-apoptotic effect [15]. In addition, recent studies have shown that Nrf2 can regulate the formation of autophagy, further promoting its protective effect [16, 17].

Based on the probucol’s characteristics of anti-oxidation, anti-apoptosis, and upregulation of protective autophagy, we attempted to give diabetic rat MSCs combined with oral probucol in order to determine its effect on the treatment of DMED and further explore the possible mechanism.

## Methods

### Experimental animals

A total of forty-eight 10-week-old Sprague-Dawley male rats weighing 260–280 g were purchased from the Animal Center of Shandong University. Animal care and treatment were approved by the Animal Care and Use Committee of Shandong University (Jinan, China). Rats were maintained in specific pathogen-free environment around 23 ± 1 °C with a 12-h light-dark cycle and supplied food and water ad libitum. All animals were adapted to the new environment for 1 week before the experiment. To establish diabetes, thirty-six rats were fasted for 12 h, then received a single intraperitoneal injection of 60 mg/kg streptozotocin (STZ, Sigma-Aldrich Chemical Co, St. Louis, MO). Twelve rats were administered vehicle only and were used as a sham group. Random blood glucose levels were monitored 72 h later after STZ or vehicle injection. Only those STZ-treated rats with random blood glucose concentrations consistently greater than16.7 mmol/L were accepted as being diabetic. These rats were divided randomly into the following 3 groups: (1) probucol (Sigma-Aldrich, St. Louis, MO, 500 mg/kg/day) was administered daily after intracavernous injection of MSCs (P + M group, *n* = 12), (2) intracavernous injection of MSCs (1 × 10^6^) in diabetic rats (M group, *n* = 12), and (3) the diabetic group (DM group, *n* = 12). Initial and final blood glucose levels and body weight of all rats were recorded.

### Isolation and cultivation of MSCs

Isolation and cultivation of bone marrow-derived stem cells were performed according to previous descriptions [18]. Typically, male Sprague-Dawley rats (80–120 g) were killed unconsciously; bone marrow tissues were washed with PBS until the bloodiness was eluted. After digestion with collagenase I and centrifugation, supernatant was discarded, and the residual cells were suspended in DMEM then cultured in humidified atmosphere and 5% CO_2_ at 37 °C for 48 h. Cells were passaged when they reached about 90% confluence, and passage 3 cells were used in vivo and in vitro experiments. To verify the cellular identity of cells, fluorescence-activated cell sorting was employed via the usage of CD90, CD29, CD34, and CD45 markers.

### The label, intracavernous injection, and observation of MSCs

Labeling of MSCs using Cm-Dil is performed under protocol. After anesthesia, the prepuce was rolled up to expose the penis; the needle was inserted by 3–4 mm under the microscope. MSCs (1.0 × 10^6^ cells in fresh PBS 100 μL) or vehicle (fresh PBS 100 μL only) was injected into the middle of the left corpus cavernosum. An elastic band was placed at the base of the penis immediately before MSC injection and was removed 3 min after the injection. Tissue was collected at 3 days, 1 week, and 2 weeks after transplantation, and frozen sections were analyzed by fluorescent microscope.

### Western blotting

Protein extraction and Western blotting were performed as previously described [19]. Tissues and cells were lysed with RIPA buffer containing protease inhibitor cocktail, and the protein concentrations of tissue lysates and cell lysates were determined by BCA assay. Samples containing 20 μg of protein were subjected to sodium dodecyl sulfate polyacrylamide gel electrophoresis and transferred to a polyvinylidene fluoride membrane. The membrane was blocked with 5% skim milk and incubated at 4 °C overnight with primary antibodies against LC3 (1:3000, Abcam), p62 (1:1000, Cell Signaling Technology), Nrf2 (1:1000, Cell Signaling Technology), HO-1 (1:1000, Abcam), Beclin1 (1:1000 Cell Signaling Technology), Bcl-2 (1:1000,Cell Signaling Technology), Bax (1:1000, Abcam), Caspase3 (1:1000, Cell Signaling Technology), and Cleaved-Caspase3 (1:2000; Cell Signaling Technology). After hybridization of secondary antibodies, the result was visualized with an LAS3000 Image Analyzer (Fujifilm, Tokyo, Japan) and data were analyzed using Multigauge software (Fujifilm).

### Immunofluorescence staining

Penile tissue was fixed in fresh 4% paraformaldehyde and then immersed in 30% sucrose in PBS overnight at 4 °C. The fixed tissues were cryoembedded in optimal cutting temperature compound (Sakura Finetek, Torrance, CA, USA) and cut into 5-μm sections before mounting on slides. After permeabilization and blocking, the slides were incubated with primary antibodies, including rabbit anti-α-smooth muscle actin (α-SMA, 1:1000; Abcam) and rabbit anti-von Willebrand factor (vWF, 1:2000; Abcam) at 4 °C overnight. At room temperature, the sections were rinsed and incubated with Alexa Fluor-594-conjugated secondary antibodies (Invitrogen, Carlsbad, CA, USA). Nuclei were stained by 4′,6-diamidino-2-phenylindole (DAPI, Invitrogen) for 5 min. Slides were visualized under a fluorescence microscope (Leica, Heidelberg, Germany).

### Masson’s trichrome stain

Masson’s trichrome stain was used to evaluate the smooth muscle cell and collagen fibril expression in cavernous tissue. Three-micrometer sections of formalin-fixed, paraffin-embedded tissues were deparaffinized in xylene (3 washes for 3 min each) and hydrated in graded ethanol to distilled water. The slides were then stained with Masson’s trichrome stain kit (Dako Sciences, Glostrup, Denmark), followed by dehydration in graded ethanol to xylene.

### Determination of cell viability using CCK8

The CCK-8 assay was used to measure MSC cell viability. MSCs (1000/well) were seeded in 96-well plates overnight. Cells were incubated with different concentrations (100, 150, 200, and 250 μM) of H_2_O_2_ for 12 h; normal culture media were used for the control group. Then, cells were first co-cultured with probucol (50, 100, and 150 μM) for 12 h and then exposed to H_2_O_2_ (250 μM) for 12 h. Normal culture media were used for the control group. At the prespecified time points, 10 μL of CCK-8 solution (DOJINDO, Kumamoto, Japan) was added to the cells. After incubation for another 4 h, the optical density (OD) values were determined at 450 nm using a microplate reader (BioTek, Winooski, VT, USA). Each group was tested in triplicate in three replicates.

### Erectile function evaluation

Measurements of maximal intracavernous pressure (max ICP) and the ratio of max ICP/mean systemic arterial pressure (MAP) were used to assess erectile function. After treatment, rats were anesthetized with 5% sodium pentobarbital. PE-50 (Intramedic; Becton Dickinson & Co., Sparks, MD) tubes were inserted into the left carotid artery of each rat to detect continuous measurement of MAP. A 26-gauge needle filled with heparin (250 U/mL) was inserted into the cavernous body of the penis to detect ICP. After finding and dissociating cavernous nerve (CN), an electrical stimulus with a frequency of 15 Hz and a pulse width of 5 ms was used to stimulate 60s. ICP and MAP were measured continuously by a BL-420V pressure transducer system (AD instrument).

### Autophagic flux measurements

To detect autophagic flux, RFP-GFP-LC3 reporter plasmid (1 μL/mL, Addgene, Cambridge, MA, USA) was transfected into MSCs using lipofectamine 2000 (Invitrogen, 11668-019) according to the manufacturer’s instructions. Then, the transfected cells were grouped into 3 groups: the sham group, H_2_O_2_ group (200 μM), and probucol group (150 μM). The cell images were obtained using Olympus FV1000 laser scanning confocal microscopy (Olympus, Tokyo, Japan).

### ROS assay

Cellular ROS levels were measured using cell permeable probe 5-(and-6)-chloromethyl-2′,7′-dichlorodihydrofluorescein diacetate (CM-H2DCFDA, Thermo Fisher, Waltham, MA, USA). Cells were loaded with 10 μM H2DCFDA in DMEM (phenol red-free) for 1 h. After washing cells twice with DMEM, fluorescence was measured with an Envision 2104 Multilabel reader (Perkin Elmer, Waltham, MA, USA).

### Statistical analysis

All experiments were repeated at least three times, and all data were presented as means ± SD. Statistical significance was analyzed by the SPSS version 22.0 software (SPSS, IL, USA). Differences between two groups were assessed using the Student *t* test, and between multiple groups using one-way ANOVA. Values of *P* < 0.05 were considered significant.

## Results

### Probucol and MSCs had no effect on body weight and blood glucose level in diabetic rats

The body weights and blood glucose levels are shown in Table 1. Compared with the sham group, the diabetic rats showed significantly higher blood glucose levels but significantly lower body weights before the experiment (*P* < 0.01). Probucol or MSC treatment or combination of probucol and MSCs did not improve the change in blood glucose level. The body weight increased for probucol and MSC intervention (*P* < 0.05).

**Table 1:** Comparisons of body weight and blood glucose in experimental animals

		Sham group	DM group	M group	P + M group
Initial	Body weight (g)	293.4 ± 21.78	305.7 ± 20.48	306.5 ± 21.05	305.9 ± 22.43
	Glucose (mmol/L)	6.18 ± 1.97	20.87 ± 1.03	20.69 ± 1.43	21.09 ± 1.13
4 weeks	Body weight (g)	414.3 ± 32.76	303.7 ± 14.38^**^	343.4 ± 17.88^#^	356.9 ± 18.51^#^
	Glucose (mmol/L)	6.26 ± 1.87	21.58 ± 2.59^**^	21.39 ± 1.98^**^	21.69 ± 2.81^**^

Data are expressed as mean ± standard deviation from *n* = 6 per group. DM group, diabetes mellitus group; M group, diabetic rats were treated by intracavernous injection of MSCs and gavage with normal saline; P + M, diabetic rats were treated by intracavernous injection of MSCs and gavage with probucol. *P < 0.05 and **P < 0.01 indicate significant difference compared with the sham group. ^#^*P* < 0.05 indicates significant difference compared with the DM group

### Identification of MSCs and the survival of MSCs

**Fig. 1:**
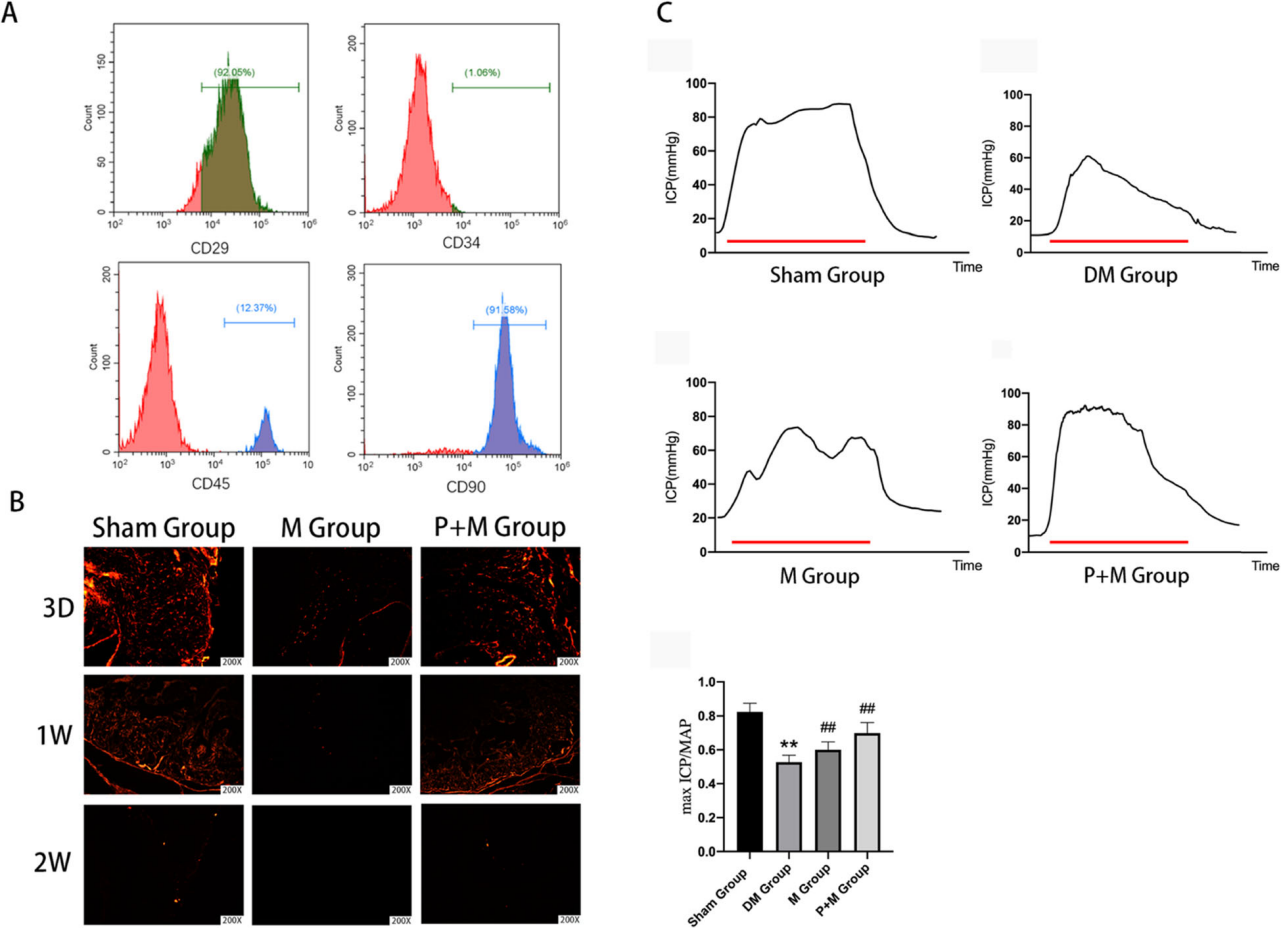
Characterization of MSCs, survival of MSCs, and erectile response after treatments. **a** The isolated cells express the MSC markers, CD29 and CD90, but do not express the hematopoietic or endothelial markers CD34 and CD45. **b** Comparisons of survival status of MSCs in different states (3 days; 1 week; 2 weeks) in frozen section. Fluorescence represents changes in the survival of the MSCs. *n* = 6 per group. **c** Treatment of probucol and MSCs improves erectile dysfunction elicited by electrical stimulation of the cavernous nerve. The colored bar denotes the 60s CN electrical stimulation. Ratios of max ICP to MAP of all the three groups were presented through bar graphs for max ICP/MAP. Data are expressed as mean ± standard deviation, *n* = 6. **P < 0.01 indicates significant difference compared with the sham group. ^##^*P* < 0.01 indicates significant difference compared with the DM group

To characterize the MSCs used in this study, we analyzed the expression of cell surface antigens, and as shown in Fig. 1a, the cells expressed markers of MSCs including CD29 and CD90, but not the hematopoietic or endothelial markers CD34 and CD45. Furthermore, we used frozen sections to detect the survival of the MSCs. As shown in Fig. 1b, after 3 days post-transplantation, more DiI-positive cells (red) were observed in the P + M group, compared with the M group, indicating that MSC survival was significantly improved due to the application of probucol. At 1 week, a large number of MSCs survived in the P + M group, while very few cells were observed in the M group. At 2 weeks, cells remained retaining in the P + M group while no cells could be observed in the M group.

### Probucol combined with MSCs improved erectile function of diabetic rats

We used maximum ICP and ICP to MAP ratios to measure changes in erectile function in rats with different interventions. As shown in Fig. 1b, rats in the DM group exerted significant decreases in both maximum ICP and ICP to MAP ratios compared to the normal rats (*P* < 0.01). MSC treatment partially ameliorated erectile dysfunction compared with diabetic rats (*P* < 0.01) while rats treated with probucol displayed significantly higher function compared with MSC-injected rats (*P* < 0.01).

### Probucol combined with MSCs improved endothelial cell function

**Fig. 2:**
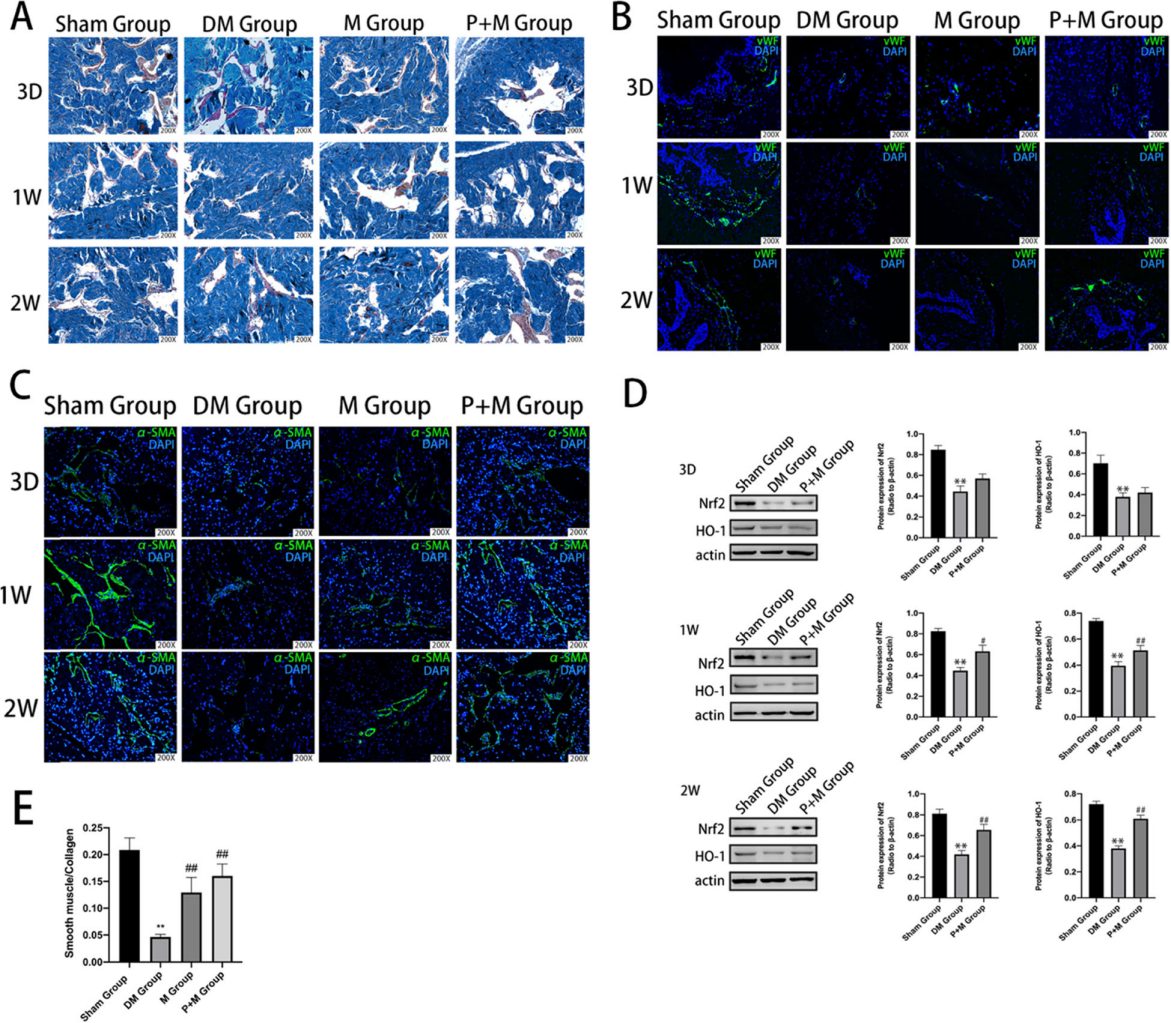
Changes of microenvironment and activation of Nrf2/HO-1 pathway in the corpus cavernosum. **a** Masson’s trichrome staining was performed to assess the corporal fibrosis level in rat cavernous of all the three groups after different treatments (the area of smooth muscle is represented by red stain, and the area of collagen by blue stain). **b** Representative immunohistochemical staining of α-SMA-positive smooth muscle (green) in the sham, DM, M, and P + M groups. **c** Representative immunohistochemical staining of vWF-positive smooth muscle (green) in the sham, DM, M, and P + M groups. **d** The expression of Nrf2 and HO-1 protein was observed at different time points among the groups. β-Actin was used as a loading control. **e** Effect of MSCs and/or probucol treatment on the ratio of smooth muscle to collagen in the corpus cavernosum in 2 weeks. Bars denote the mean densitometry ratio between smooth muscle content and collagen content per field. **P < 0.01 indicates significant difference compared with the sham group. ^#^*P* < 0.05 and ^##^*P* < 0.01 indicate significant difference compared with the DM group

Immunofluorescence staining analysis of α-SMA expression showed a significant decrease of smooth muscle content in diabetic rats (Fig. 2c). The corpus cavernosum was evaluated for the smooth muscle/collagen ratios on slides stained with Masson’s trichrome. As shown in Fig. 2a, both MSC and probucol treatments restored the smooth muscle content. The smooth muscle/collagen ratio in different groups is shown in Fig. 2e. Surprisingly, probucol treatment displayed a better recovery with higher smooth muscle density compared to the DM Group (*P* < 0.01). The von Willebrand factor (vWF)-positive area of the endothelium content in the DM group was significantly lower than in the sham group (Fig. 2b). After IC injection of MSCs, the endothelium content was increased to some degree. After probucol treatment, the endothelium in cavernous tissue was better restored compared with MSC-injected rats.

### Probucol activated Nrf2/HO-1 antioxidant stress pathway

In Fig. 2d, Western blotting results showed that diabetes mellitus caused significant decreased expression of Nrf2 and HO-1 in the corpus cavernosum (*P* < 0.01). Rats in the P + M group showed a significant recovery of Nrf2 (*P* < 0.05) and HO-1 expression (*P* < 0.01) in 1 week. Obviously, after 2 weeks of treatment, probucol’s intervention increased more Nrf2 and HO-1 expression compared with the DM group (*P* < 0.01).

### Probucol reduced the level of ROS, increased cell viability, and activated Nrf2/HO-1 antioxidant stress pathway in H_2_O_2_-treated MSCs

**Fig. 3:**
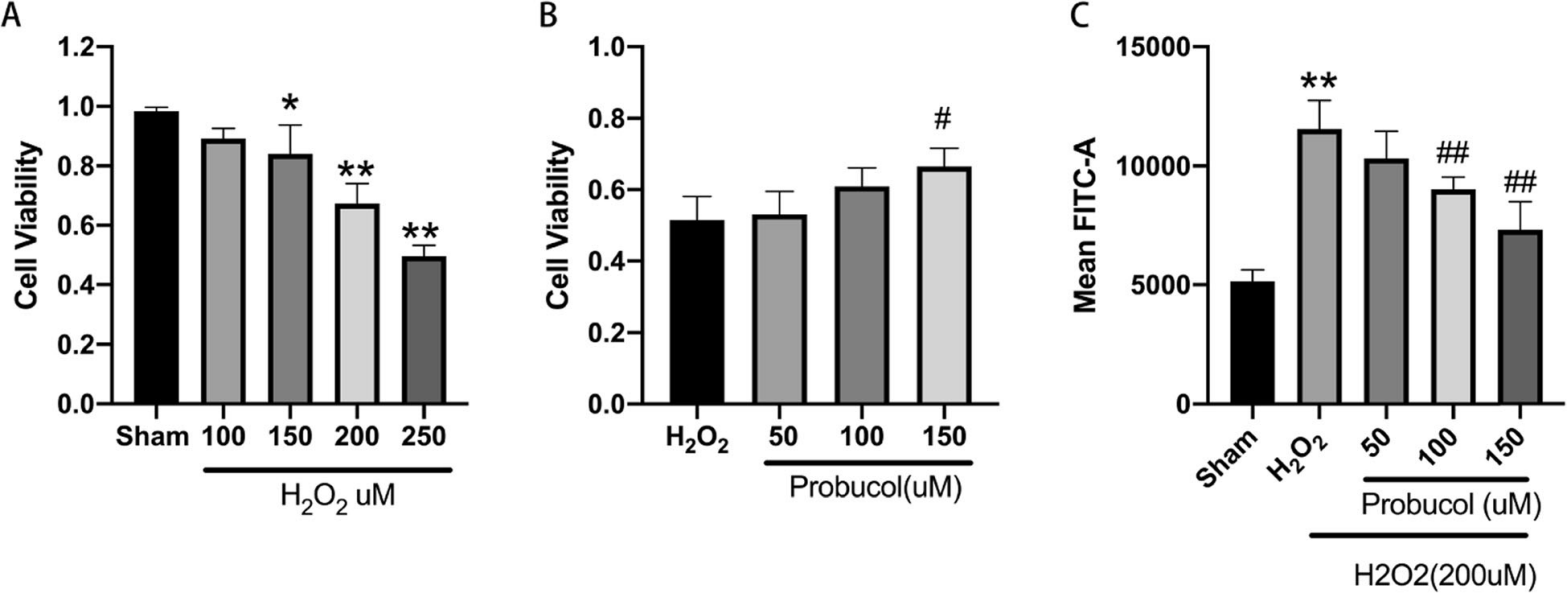
Effect of H_2_O_2_ and probucol on MSC viability and ROS. **a** H_2_O_2_ treatment inhibits the viability of MSCs in a concentration-dependent manner. IC50 value was calculated using CCK8 assay in triplicates; subIC50 dose of 200 μM was used for further experiences. **b** Probucol’s intervention could reverse the injury of H_2_O_2_ to MSCs, and was statistically significant at the concentration of 150 μM. **c** Probucol reduces ROS levels in MSCs. Intracellular ROS in the normal group, the oxidative stress group, and the probucol group were measured by flow cytometry before histogram was made. *P < 0.05 and **P < 0.01 indicate significant difference compared with the sham group. ^#^*P* < 0.05 and ^##^*P* < 0.01 indicate significant difference compared with the H_2_O_2_ group

To investigate the effect of probucol on MSCs under oxidative stress, we used CCK8 to detect the survival of MSCs under different conditions. In Fig. 3a, CCK8 results indicated that H_2_O_2_ induced a dose-dependent decrease of cell viability. MSC cell viability was reduced to 50% at 12 h by 200 μM H_2_O_2_, a concentration below its IC50 value (250 μM), which was selected as a standard concentration for subsequent experiments. As shown in Fig. 3c, after exposure to H_2_O_2_, ROS production in MSCs increased. Compared to the H_2_O_2_ group, cell viability was significantly increased (Fig. 3b), and ROS production was dramatically decreased in the H_2_O_2_ + probucol (150 μM) group in a dose-dependent manner (Fig. 3c).

### Probucol decreases apoptosis in MSCs

**Fig. 4:**
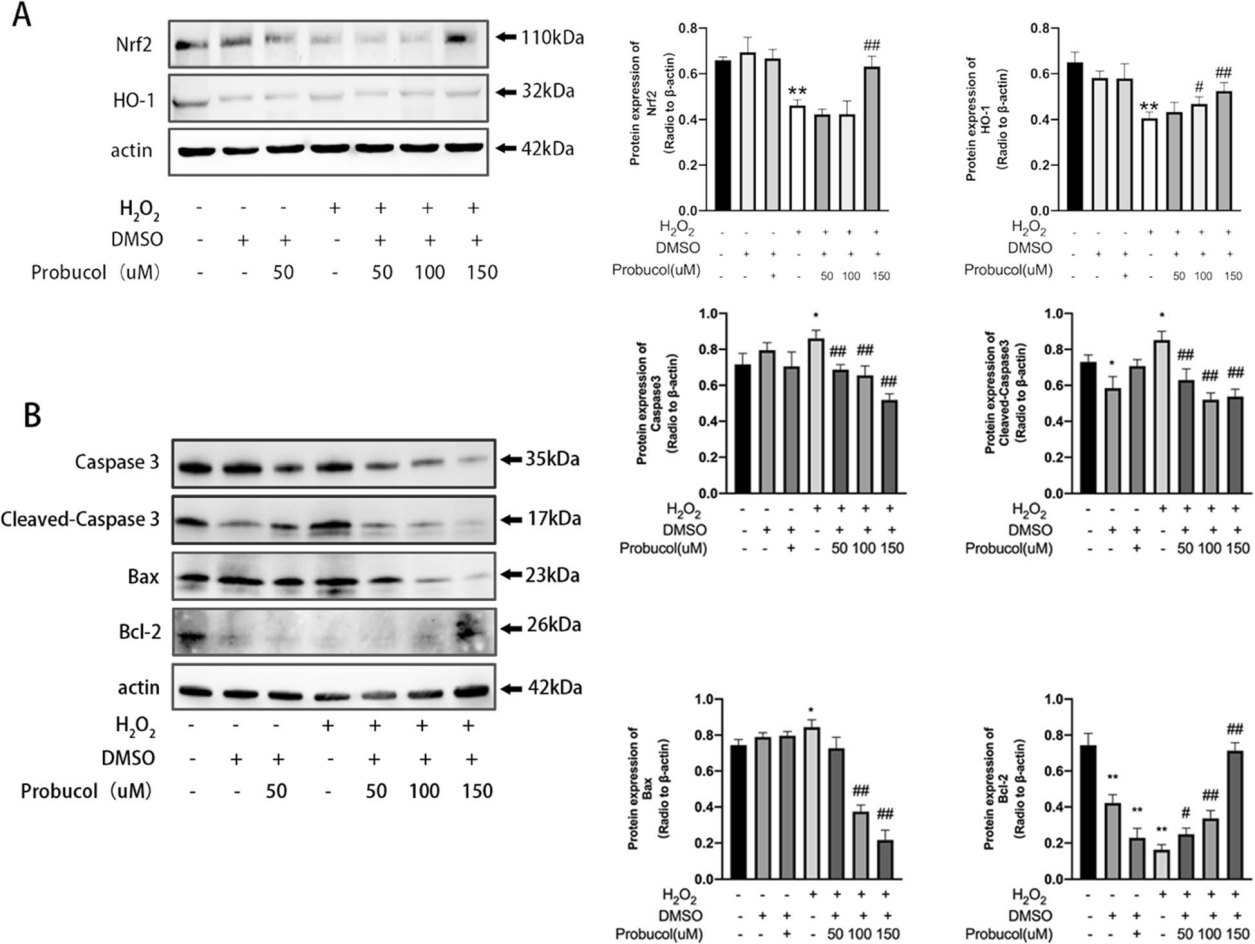
Probucol activates Nrf2/HO-1 pathway and reduces apoptosis in MSCs. **a** Representative images of Western blotting of Nrf2 and HO-1 in MSCs treated with different concentrations of probucol. β-Actin was used as a loading control. **b** Probucol reduced H_2_O_2_-induced apoptosis in MSCs. Representative images of Western blotting of Caspase3, Cleaved-Caspase3, Bax, and Bcl-2 in MSCs treated with different concentrations of probucol. β-Actin was used as a loading control. *P < 0.05 and **P < 0.01 indicate significant difference compared with the sham group. ^#^*P* < 0.05 and ^##^*P* < 0.01 indicate significant difference compared with the H_2_O_2_-treated group

Figure 4a suggests that under H_2_O_2_-induced oxidative stress, Nrf2 and HO-1 expression were significantly decreased (*P* < 0.01), while probucol could significantly upregulate Nrf2 and HO-1 expression in a dose-dependent manner, reaching a peak at 150 μM (*P* < 0.01). Besides, increased ratio of pro-apoptotic Bax to anti-apoptotic Bcl-2 induces mitochondrial cytochrome C release, a hallmark of apoptosis. We used Western blotting to detect the apoptosis of MSCs. The results in Fig. 4b exerted that H_2_O_2_ significantly increased the expression of Bax, Caspase3, and Cleaved-Caspase3 and decreased the expression of Bcl-2. The results indicated that H_2_O_2_ induced apoptosis of MSCs. The expression of Bax, Caspase3, and Cleaved-Caspase3 decreased, and the expression of Bcl-2 increased in a dose-dependent manner because of probucol’s intervention at 150 μM (*P* < 0.01). These results suggested that probucol reduced H_2_O_2_-induced apoptosis.

### Probucol enhanced MSCs’ protective autophagy

**Fig. 5:**
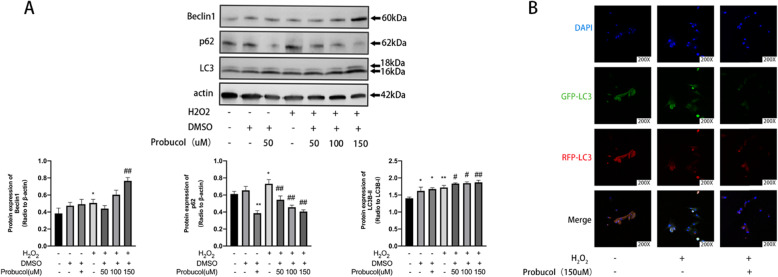
Probucol activates autophagy of MSCs and does not block autophagy flux. **a** MSCs were exposed to probucol (0, 50, 100, 150 μM) for 12 h in the presence of H_2_O_2_. Western blotting analysis for expression levels of Beclin1, P62, and LC3B-II/I. The protein band intensities were normalized to β-actin, expressed relative to control. **b** MSCs were transfected with RFP-GFP-LC3. The cells were then incubated in the absence or presence of probucol (150 μM) for 12 h. Fluorescence microscopy images of MSCs were obtained by monitoring GFP-LC3 puncta (green fluorescence), and the lysosomes were stained with RFP-LC3 (red fluorescence). Insets show an enlarged image of the merged field of interest

Western blotting results in Fig. 5a showed that probucol could induce the expression of Beclin1 and LC3II in a dose-dependent manner, peaking at 150 μM (*P* < 0.01). At the same time, the expression of P62 decreased continuously (*P* < 0.01). In order to further determine whether probucol had a negative impact on autophagy and lysosome, we used RFP-GFP-LC3-transfected cells to mark autophagic vacuoles (yellow) and analyzed cell image by confocal microscope after intervention. As shown in Fig. 5b, after autophagy-lysosomal fusion, GFP was released from RFP-GFP-LC3 and degraded in lysosomes. Therefore, autolysosome would lead to the formation of fluorescence from yellow to red. In MSCs treated with probucol, autophagy lysosomes were observed, showing an increase in red spots. These results suggested that probucol could enhance the protective autophagy of MSCs by facilitating autophagosome-lysosome fusion.

## Discussion

In recent years, stem cell transplantation therapy has been regarded as a promising weapon against ED. Mesenchymal stem cells (MSCs) receive particular concerns as the result of their featured biological benefits, including abundance of autologous sources, ease of isolation, and expanding [20]. Meanwhile, many studies have shown that MSC transplantation had significant effect on a variety of diseases, including DMED [20–22]. In this study, we focused on the effect of the combination therapy and the impact of probucol on MSCs under oxidative stress. Our results indicated that the combined therapy could better improve the erectile function of diabetic rats, which might be related to the longer survival time of MSCs in the corpus cavernosum of diabetic rats. What is the mechanism? We thought it might be related to probucol-induced decrease in oxidative stress, increase in antioxidation and protective autophagy, and inhibition of apoptosis triggered by activation of the Nrf2 pathway.

Probucol diminished the oxidative stress level in the corpus cavernosum of diabetic rats. Nrf2-mediated antioxidant response to oxidant injuries has been detected in the corpus cavernosum from diabetic rats, suggesting that diabetic-related Nrf2 dysfunction increases vulnerability of the corpus cavernosum to oxidative stress-induced damage [11]. Nrf2 exerts its antioxidant activity by upregulating the expression of some effector enzymes, including HO-1 [23]. Our previous studies confirmed that activation of Nrf2/HO-1 pathway could reduce ROS production, which decreased the oxidative stress level of penile cavernous body in diabetic rats [11]. The results of current study showed the amounts of Nrf2 and HO-1 were significantly reduced in the penile cavernous body of diabetic rats, while probucol’s intervention reversed that and improved erectile function of diabetic rats. Therefore, it was proved that probucol could alleviate the hyperoxidative stress of penile cavernous body in diabetic rats and provide a good microenvironment for MSC transplantation.

Probucol enhanced self-antioxidant ability of MSCs through Nrf2. In in vitro experiments, we studied the effect of probucol on MSCs under oxidative stress. The secondary injury following H_2_O_2_ represents consecutive pathological processes including oxidative stress and apoptosis [24]. Oxidants and their dericatives generated by H_2_O_2_ enhance the generation of ROS and exhaust antioxidant defense enzymes. Level of expression of CCK8 showed probucol reverted the deleterious effect of H_2_O_2_ on MSC viability. Meanwhile, the activation of Nrf2 by probucol was dose-dependent and reached a peak at 150 μM. It is well documented that HO-1 is positively regulated by Nrf2 [25] which was confirmed by our results that the expression of HO-1 increased with the increase of probucol in a dose-dependent manner. As an anti-oxidative stress protein, HO-1 causes hemoglobin decomposition to form ferrous iron, carbon monoxide, and biliverdin, which resist the damage caused by oxidative stress [26]. Therefore, these data indicated that probucol enhanced MSCs’ ability to resist oxidative stress by activating Nrf2/HO-1 pathway.

Probucol enhanced the protective autophagy of MSCs. It is not completely clear how autophagy is regulated by Nrf2, which do not get around P62. P62 is a lysosome protease substrate and has the dual binding sites of ubiquitin chains and LC3. P62 combines with LC3 through LC3 interaction area (LIR). Ubiquitin chains through UBA (related to ubiquitin) structure domain combine with LC3, so as to activate autophagy [27]. Thus, stimuli such as oxidative stress have been shown to induce autophagy and degradation of P62, and to reduce intracellular P62 levels [28]. Puissant and his colleagues suggested Nrf2 specificity to combine in P62 promoter of the antioxidant response element, promote the expression of P62, then combine with LC3, and activate autophagy [29]. Chunjuan Song and colleagues found that autophagy under oxidative stress was thought to be self-regulation of cells against harmful stimuli [30]. Our results suggest that probucol could increase Beclin1 and LC3-II levels and decrease the levels of P62 under oxidative stress. Moreover, autophagy flux suggested that probucol intervention did not inhibit autophagy flux, thus leading to autophagy accumulation. Double-labeled immunofluorescence analysis suggested that H_2_O_2_ inhibited the autophagy level of MSCs, resulting in GFP-LC3 accumulation in the cytoplasm. Probucol reversed this phenomenon by increasing expression of RFP-LC3, while activated autophagy and autophagy flux were not inhibited. These results suggested that probucol intervention enhanced MSCs’ resistance to oxidative stress by increasing protective autophagy. Further studies are needed to clarify how Nrf2 regulates autophagy.

Probucol inhibited the apoptosis of MSCs under oxidative stress. Oxidative stress increases mitochondrial depolarization and Ca^2+^ entry, then induces mitochondrial release of cytochrome C into the cytosol and sequential activation of Caspase3 and Caspase9, leading to apoptosis [31]. Previous experiments have shown that Caspase3 is a necessary pathway for the cascade of apoptotic proteases [32], and mitochondrial Bcl-2 family is the most important regulatory factor in the endogenous apoptotic pathway [33]. Apoptosis can be initiated through P53-induced activation of Bax in a caspase-dependent manner [34]. Our results showed that the expression of Bcl-2 was downregulated while Bax was upregulated in MSCs with H_2_O_2_ intervention. H_2_O_2_ intervention significantly upregulated the expression of apoptosis-related proteins Caspase3 and Cleaved-Caspase3, suggesting that H_2_O_2_ induced MSC apoptosis through mitochondrial Caspase3 pathway. Oral treatment with probucol reversed the above changes, leading to the upregulation of Bcl-2 and the downregulation of Bax, reducing downstream expression of Caspase3 and Cleaved-Caspase3. These results suggested that H_2_O_2_ could break the balance between Bcl-2 and Bax in the Bcl-2 gene family, leading to a decrease in the Bcl-2/Bax ratio of MSCs under oxidative stress, thereby activating the mitochondrial Caspase3 pathway and promoting apoptosis. The possible mechanism was that probucol could activate Nrf2, thus upregulating the transcription of Bcl-2 gene. Furthermore, Bcl-2 mediated by Nrf2 lead to the decrease of Bax and apoptosis [35].

Furthermore, tissue structure was analyzed by Masson’s trichrome staining and immunology staining with vascular endothelial marker (vWF) and vascular smooth muscle marker (α-SMA). Our previous studies have shown that probucol improves endothelial function and reduces fibrosis [36]. Our results shown that compared with MSCs alone, combination therapy increased the expression of vWF and α-SMA. The above physiological processes worked together to restore the vascular endothelial function of penile cavernous body and reduce fibrosis. These changes provided the structural basis for the improvement of ED. The mechanism of structural change in the corpus cavernosum was not explored in this study.

## Conclusion

In conclusion, we demonstrate that probucol enhances the therapeutic efficiency of MSCs in the treatment of DMED by prolonging their survival time via Nrf2 pathway. These effects appear to be mediated through improved microenvironment of transplanted MSCs, by increasing their self-antioxidant ability, increasing protective autophagy, and diminishing apoptosis.

## Data Availability

All data generated or analyzed during this study are included in the published article.

## Abbreviations

MSCs: Mesenchymal stem cells
DMED: Diabetic mellitus-induced erectile dysfunction
IC: Intracavernosal
ED: Erectile dysfunction
DM: Diabetes mellitus
PDE5i: Phosphodiesterase type 5 inhibitors
ROS: Reactive oxygen species
Keap1: Kelch sample ECH associate protein 1
STZ: Streptozotocin
DMEM: Dulbecco’s modified Eagle’s medium
PBS: Phosphate buffer saline
α-SMA: α-Smooth muscle actin
vWF: von Willebrand factor
OD: Optical density
CN: Cavernous nerve
ICP: Intracavernous pressure
MAP: Mean arterial blood pressure
LIR: LC3 interaction area
