# Correction to: Probucol enhances the therapeutic efficiency of mesenchymal stem cells in the treatment of erectile dysfunction in diabetic rats by prolonging their survival time via Nrf2 pathway

**DOI:** 10.1186/s13287-022-02727-0

**Published:** 2022-01-31

**Authors:** Haoran Wang, Keqin Zhang, Zheng Ruan, Dingqi Sun, Hui Zhang, Guiting Lin, Liangliang Hu, Shengtian Zhao, Qiang Fu

**Affiliations:** 1 Department of Urology, Shandong Provincial Hospital, Cheeloo College of Medicine, Shandong University, Jingwuweiqi Road 324#, Jinan, 250021 Shandong People’s Republic of China; 2 Department of Urology, Shandong Provincial Hospital Affiliated to Shandong First Medical University, Jinan, 250021 Shandong People’s Republic of China; 3 Tai’an City Central Hospital, Tai’an, 271000 People’s Republic of China; 4 Knuppe Molecular Urology Laboratory, Department of Urology, School of Medicine, University of California, San Francisco, CA USA; 5 Department of Urology, Shandong Zaozhuang Municipal Hospital, Zaozhuang, 277000 People’s Republic of China

## Correction to: Stem Cell Research & Therapy (2020) 11:302 https://doi.org/10.1186/s13287-020-01788-3

Following the publication of this article [1], the authors notice the immunohistochemical staining of α-SMA in Fig. 2 contains incorrect images. The authors put the same picture in 2W (Sham Group) and 1W (P + M Group) by mistake when they assembled the figure. After checking the original data, the authors realized that there was an error with the image selection during manuscript preparation. In addition, the Fig. 2 legend also needs correction, that Fig. 2b is vWF staining, while Fig. 2c is SMA staining.

**Fig. 2:**
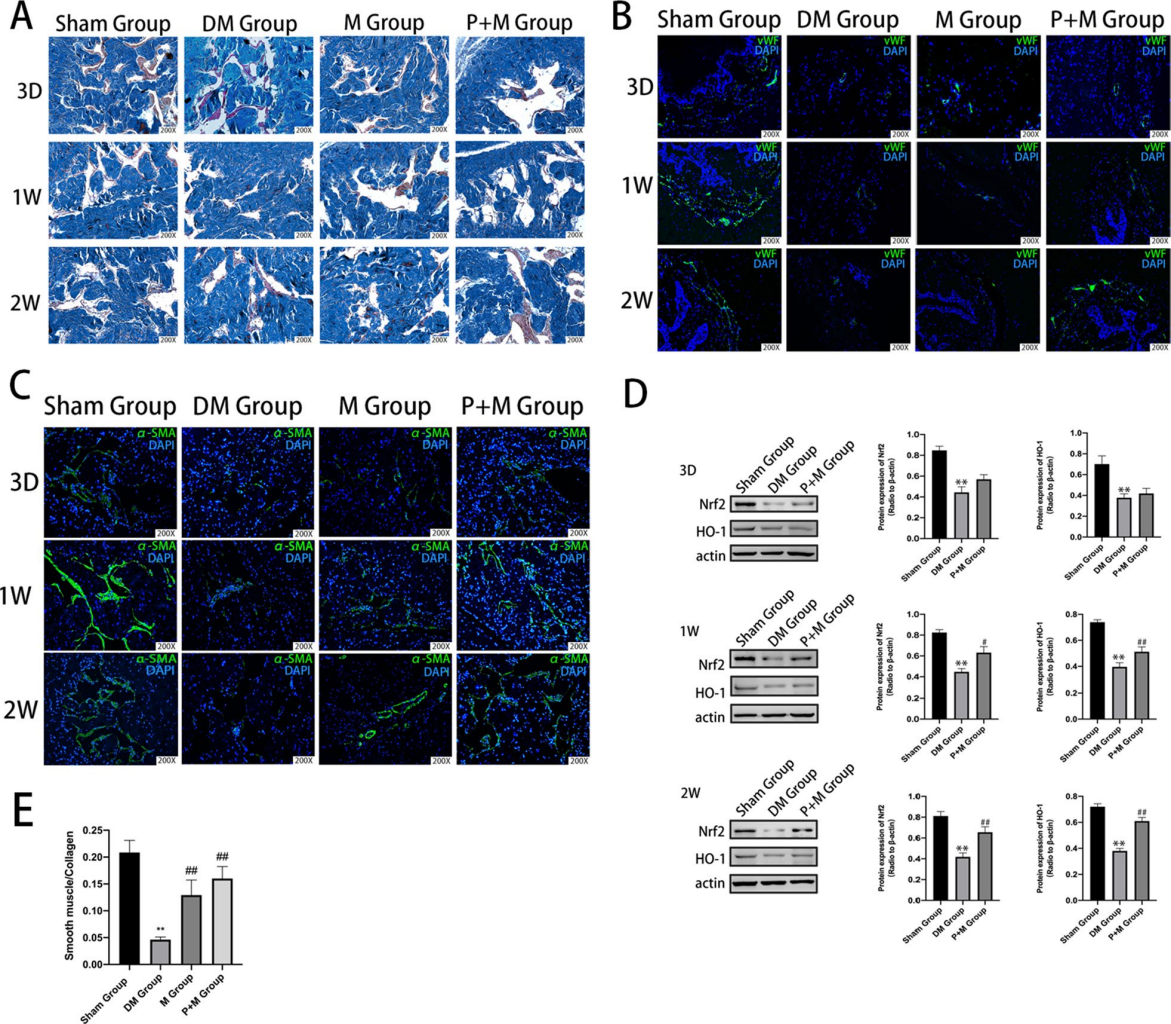
Changes of microenvironment and activation of Nrf2/HO-1 pathway in corpus cavernosum. **A** Masson’s trichrome staining was performed to assess the corporal fibrosis level in rat cavernous of all the three groups after different treatment (the area of smooth muscle is represented by red stain and the area of collagen by blue stain). **B** Representative immunohistochemical staining of vWF-positive smooth muscle (green) in Sham, DM, M, P + M Groups. **C** Representative immunohistochemical staining of α-SMA-positive smooth muscle (green) in Sham, DM, M, P + M Groups. **D** The expression of Nrf2 and HO-1 protein was observed at different time points among the groups.β-Actin was used as a loading control. **E** Effect of MSCs and/or probucol treatment on the ratio of smooth muscle to collagen in the corpus cavernosum in 2 weeks. Bars denote the mean densitometry ratio between smooth muscle content and collagen content per field. **p < 0.01 indicates significant difference compared with Sham Group. ^#^*p* < 0.05 and ^##^*p* < 0.01 indicate significant difference compared with the DM Group

The correct Fig. 2 and the figure legend have been included in this correction.

The correction will not affect the result and scientific conclusion of the manuscript. The authors would like to apologize for any inconvenience caused.
